# The Effect of Breathing Exercise Using Bubble Blower on Anxiety and Pain during Inferior Alveolar Nerve Block in Children Aged 7 to 10 Years: A Crossover Randomized Clinical Trial

**DOI:** 10.1155/2022/7817267

**Published:** 2022-01-17

**Authors:** Zahra Bahrololoomi, Tahereh Sadeghiyeh, Maedeh Rezaei, Nahid Maghsoudi

**Affiliations:** ^1^Department of Pediatric Dentistry, Social Determinants of Oral Health Research Center, Dentistry Faculty, Shahid Sadoughi University of Medical Sciences, Yazd, Iran; ^2^Child and Adolescent Psychiatrist, Researcher of Center of Addiction and Behavioral Sciences, Shahid Sadoughi University of Medical Sciences, Yazd, Iran; ^3^Department of Pediatric Dentistry, Dentistry Faculty, Shahid Sadoughi University of Medical Sciences, Yazd, Iran

## Abstract

**Introduction:**

The aim of this study was to evaluate the effect of breathing exercise using bubble blower on anxiety and pain during inferior alveolar nerve block (IANB) in children aged 7 to 10 years.

**Materials and Methods:**

In this randomized crossover clinical trial, thirty-five children with moderate to severe anxiety requiring bilateral pulp therapy of mandibular primary molars were enrolled. Based on random lists, 18 children received the BE + IANB and 17 children received a routine IANB at the first session. This trend became reverse at the second visit for each child. Anxiety was measured using Facial Image Scale (FIS), blood pressure, and pulse rate. Face Leg Activity Cry Consolability (FLACC) scale and Wong–Baker Facial Pain Scale (WBFPS) were used for pain measurement. The Paired Samples Test, Wilcoxon Signed Rank Test, and Interclass Correlation Coefficient were used for data analysis.

**Results:**

The means of FLACC, WBFPS, FIS, blood pressure, and pulse rate were higher at the control visit. However, these differences were statistically significant only for FLACC scale and WBFPS (*P* value <0.05). In subgroup analysis, only girls and children without any previous dental treatment showed significant differences in FLACC scale and WBFPS between the control and bubble blower side (*P* value <0.05).

**Conclusion:**

Breathing exercise using a bubble blower may be an efficient distraction and relaxation method to decrease pain of 7- to 10-year-old children with moderate to severe anxiety during inferior alveolar nerve block. However, anxiety levels were lower when applying BE, and the differences were not statistically significant.

## 1. Introduction

Pain and anxiety control for children is an essential component of pediatric dental care. High levels of anxiety and fear might exhibit increased sensitivity and responsiveness to painful stimuli. Fearful children tend to avoid dental care and have poor oral health followed by increased caries incidence and dental emergencies and decreased oral health-related quality of life [1]. Patients with excessive dental fear and anxiety might struggle with sleep disorders, negative thoughts, and low self-confidence [2]. A study by Akbay Oba et al. showed significant correlations between dental fear and incidence of dental caries; higher scores of CFSS-DS were accompanied by higher values of dmfs/DMFS [3]. Needle phobia is the most common dental fear among children and IANB has been graded to be the most painful local anesthesia [4]. Local anesthesia injection to a child with high levels of anxiety has been found as the most stressful procedure without considering the age, gender, or years of professional experience for general dentists and pedodontists [5]. Single buccal infiltration, Wand computerized delivery system, and intraosseous anesthesia systems have been introduced as alternatives for IANB. The new techniques seek expensive equipment without any enhanced efficacy compared to traditional techniques [6]. Buccal infiltration for mandibular primary molars is not an effective anesthetic technique for invasive procedures like pulp therapy or extraction [7].

General anesthesia or sedation can be good choices for managing severely anxious children or patients who are not able to cooperate efficiently, but children with lower levels of anxiety and fear should be managed with traditional behavioral guidance techniques [8]. Distraction strategies have been effective methods for stress and anxiety reduction during local anesthesia administration. Distraction may reduce the pain perception by involving the child's focus and attention. Active forms of distraction actively involve the child resulting in more efficacy compared to passive distraction [9]. Relaxation BE is an active distraction which induces vagus nerve stimulation followed by cortisol reduction and antidepressant neurotransmitters secretion such as serotonin, and also BE distracts the child's attention from the painful provocations; these mechanisms alleviate the patient's anxiety and pain perception [10, 11]. The use of a bubble blower for BE turns this technique to a play therapy.

Play therapy is a dynamic interpersonal relationship between the child and the therapist which helps the child to control negative emotions and stresses. Play therapy can act as a coping mechanism in dealing with challenging situations so the child feels the sense of control over the stressful event [12]. Moreover, BE can be considered as a behavioral coping strategy because of physical involvement of the body and this type of coping strategy has been more effective in pain score reduction in studies involving venipuncture [13].

BE has effectively relieved acute pains such as vaccination or cryotherapy of dermal warts and chronic pain of children with cancer or epidermolysis bullosa [14, 15]. Studies of pediatric dentistry have reported significant reduction in pain scores during maxillary infiltration and IANB in BE group compared to the control group [16, 17].

In the breathing exercise technique, the child inhales deep breaths from the stomach and exhales very slowly. This exercise is performed 4–5 times a day for one week at home before the dental visit. At the dental session, the exercise is repeated during the local anesthesia injection. It may help the child to control his/her breath and get relaxed during the injection. This method is inexpensive, simple, and quick to administer. The objective of the present study was the evaluation of the effect of breathing exercise using bubble blower on anxiety and pain during inferior alveolar nerve block in children aged 7 to 10 years.

## 2. Materials and Methods

### 2.1. Study Design and Participants

The present randomized crossover clinical trial has been carried out between November 2020 and March 2021. The study participants were 7- to 10-year-old children referring to Pediatric Dentistry Department of Shahid Sadoughi University of Medical Sciences. The trial has been approved at the Ethics Committee of the university by IRB code of IR.SSU.REC.1399.024. A dentist who was one of the members of the ethical committee of the university supervised the study progress and ethical considerations. He supervised informed consent acquisition from all patients, study progression according to the proposed protocol (such as sample size, participants recruitment, and interventions), and adequate treatment for all patients by unexpected visits to the pediatric clinic. The study protocol has been verified by the Iranian Registry of Clinical Trials with a registration ID of IRCT20191002044953N2.

#### 2.1.1. Inclusion Criteria

1. Healthy children aged 7- to 10-year-old with moderate to severe anxiety (score 3 to 5 of FIS)
2. Requiring bilateral pulp therapy of primary mandibular molars
3. No allergy to anesthetic drugs
4. Incapability of communication in Farsi
5. Not seeking emergency cares
6. No major psychological disorders or learning disability or parental anxiety

#### 2.1.2. Exclusion Criteria

1. Uncooperative behavior of patient (definitely negative, Frankl's behavior rating)
2. Use of analgesic before treatment
3. Teeth with necrotic pulp or acute abscess
4. Uncooperative parents

### 2.2. Sample Size Determination

The sample size was calculated at 95% confidence interval and 80% power of the study, based on a standard deviation of 0.6 for FIS (as the primary endpoint) and a difference of 0.4 in the mean FIS between visits. Thus, the sample size was set at a minimum value of 35.

### 2.3. Interventions

A number of 104 children who referred to pediatric dental clinic of Shahid Sadoughi University of Medical Sciences were screened for patients' recruitment. After taking medical and dental history as well as clinical evaluation and radiographic diagnosis, the patients received fluoride therapy and tell-show-do for behavior guidance. A single investigator carried out all the procedures of the trial. The patients were asked to rate their anxiety according to FIS; the scale comprises five drawings of faces denoting emotions of very happy to very unhappy, scored 1 to 5. The children were asked to indicate the face which they felt most like while sitting on the dental chair [18]. The chosen FIS score of each child was recorded. If the child met the inclusion criteria of the trial, the study protocol was fully described to the patient's guardians and the patient was entered into the trial only after the informed consent was signed.

Children were randomized into two groups. The first group received the instruction of BE as well as a commercial bubble blower immediately after inclusion to the trial. The patients and their parents were asked to practice 4–5 times daily for 7 days with the bubble blower and record every practice in a chart prepared in advance by the investigator. The second group did not receive neither BE instruction nor the bubble blower at this visit. The first treatment sessions for both groups were scheduled for a week later. In group 1, the child was asked to breathe during IANB injection exactly the same as the BE they practiced at home. After 7 days of washout period, the patient received pulp therapy of the contralateral molar with a routine IANB without any BE. In group 2, a routine IANB was delivered to the patient at the first session of pulp therapy, and at the end of this appointment, the children and parents were instructed to perform BE similar to the first group. After a week of practicing, patients returned for the second pulp therapy so that they repeated BE during IANB injection.

During BE, the children should inhale deep breaths from the stomach and exhale very slowly to make the largest bubbles they can. The parents supervised and helped the children.

For IANB injection for both groups, a 20% benzocaine topical anesthetic gel (benzocaine, Beutlich, USA) was applied on isolated mucosal surface for at least 1 minute. A long 27-gauge needle (Septoject, Septodont Inc., New Castle, DE, USA) was inserted between the internal oblique ridge and the pterygomandibular raphe depositing 1.8 ml of 2% lidocaine with 1 : 10000 epinephrine (Persocaine, DarouPakhsh Co., Tehran, Iran).

A hidden camera recorded the child's behavior and reactions before anesthetic gel application until the IANB injection was done. Therefore, the observers could rate FLACC score more precisely by moving the video back and forth [19].

A digital wrist blood pressure monitor (Beurer PC30 Wrist Blood Pressure Monitor, Beurer GmbH, Soflinge Str., Germany) measured preinjection and postinjection blood pressure and pulse rate. Postinjection measurements of blood pressure and pulse rate were administered immediately after IANB delivery [20]. FIS was evaluated after injection by asking the child to choose a picture he/she feels most likely. Then, the child was asked to indicate the degree of perceived pain by selecting one of the six faces of WBFPS [21].

Pulp therapy followed by amalgam filling or stainless steel crown placement was done for all patients at both visits.

### 2.4. Blinding

It was not possible to blind neither the investigators nor the patients due to the nature of the intervention.

### 2.5. Randomization and Allocation Concealment

A computer-generated random block design was prepared by a statistics consultant. Each block represented a patient and consisted of two quadrants of the mandible (A: the trial side, IANB + BE, and B: the control side, routine IANB), and block randomization allowed equal distribution of mandibular quadrants into two groups. A second simple random sequence was generated to determine which side will be treated at the first visit (A: right, B: left). The sequence of block randomized list was entered into 35 sheets of standard sized. Then, a black paper was placed on top of each sheet and both sheets were placed into an envelope. All 35 blocks were completed similarly. The envelopes were mixed thoroughly in a plastic container, and then the envelopes were marked sequentially from 1 to 35. Similarly, the second sequence was written on 35 sheets. All 35 envelops were shuffled in another plastic container and sequentially numbered from 1 to 35. The envelopes were placed in a numerical order into each container and were opened sequentially by the patients after patients' recruitment at the examination session [22].

### 2.6. Statistical Analysis

Collected data were analyzed using SPSS 25 (SPSS Inc., Chicago IL, USA). Interclass Correlation Coefficient assessed the consistency and agreement of investigators scoring the FLACC scale. To find the significance difference in parametric data (blood pressure and pulse rate) between visits, Paired Samples Test was used. To find the significance in nonparametric data (FIS, WBFPS, and FLACC), Wilcoxon Signed Rank Test was used. To find out the effect of other factors such as gender or history of previous dental treatment on the differences between visits, data were subgrouped into four subgroups and all statistical analyses were individually performed for each subgroup. The significance level was set at the probability value of 0.05.

## 3. Results

### 3.1. General Characteristics

**Figure 1:**
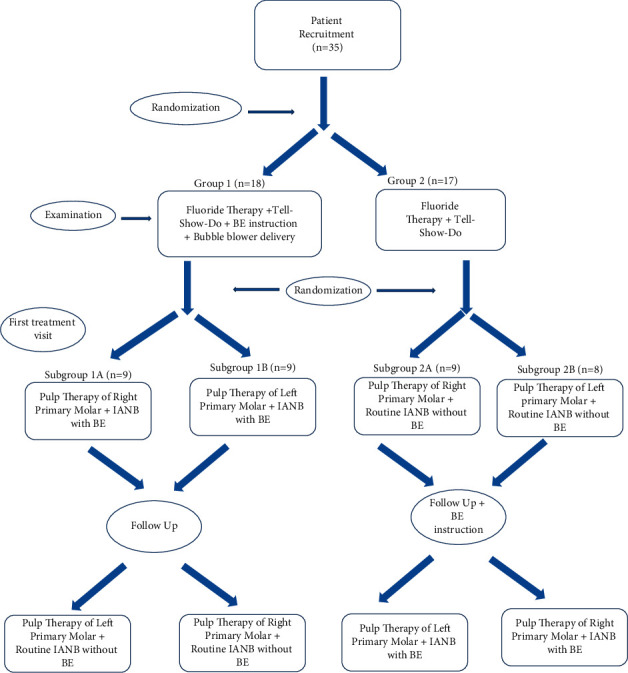
Flowchart of the trial.

After screening 120 children who referred to Pedodontics Department of Shahid Sadoughi University of Medical Sciences, 35 children were enrolled into the trial. The patients comprised 18 (51%) girls and 17 (49%) boys. Also, 16 (45%) patients reported previous dental treatment. The mean age of participants was 8.32 ± 0.57 years with a range of 7 to 9.5. Of the total participants, 17 (49%) received the routine IANB injection at the first visit and IANB + breathing exercise at the second visit; this trend was reverse for the other 18 patients (51%). The flowchart of the trial is presented in Figure 1.

### 3.2. Clinical Parameters

Differences of preinjection and postinjection blood pressure revealed abnormal distribution based on Kolmogorov–Smirnov test. Thus, Wilcoxon Signed Rank Test was used for analysis of these parameters. Other data related to blood pressure and pulse rate were normally disturbed and analyzed by Paired Samples Test. The means of systolic and diastolic blood pressure and pulse rate, measured after injection, were higher at the control visit compared to the bubble blower visit. However, Paired Samples Test did not show any statistically significant differences (*P* value >0.05).

The mean of differences of preinjection and postinjection blood pressure and pulse rate was also insignificantly higher for the control side (*P* value >0.05) (Table 1).

Interclass Correlation Coefficient showed reliable measurements of observers with a correlation coefficient of 0.833 (*P* value ≤0.001). There were lower mean values of FLACC, WBFPS, and FIS scales in bubble blower side compared with the control side. Wilcoxon Signed Rank Test showed significant differences between the test and the control side in terms of WBFPS and FLACC scale (*P* value <0.05) (Table 2). There were no significant differences in the mean values of FIS between visits (*P* value >0.05).

Subgroup analysis for gender and history of previous dental treatments did not show any significant differences in any parameters between bubble blower and the control visits for boys and children with a previous dental treatment (*P* value >0.05).

However, girls and children without previous dental treatment showed significant differences in WBFPS, FLACC1, and FLACC2 (*P* value <0.05). No significant differences were found in other data in girls or children without previous dental treatment (*P* value >0.05).

No adverse events were reported following administration of either IANB injection or pulp therapy and restorative treatments.

## 4. Discussion

The present randomized crossover clinical trial evaluated efficacy of BE using a commercial bubble blower on anxiety and pain during IANB injection in 7- to 10-year-old children. Injection produces the greatest negative responses in pediatric dental care and continuous exposure to injection procedure sensitizes the children and causes increasingly negative responses [4]. In the present study, IANB injection at the first appointment, which is rated by the children as the most painful dental anesthesia, might have a negative impact on children' behavior at the second visit. Also, there was a possibility that the injection technique at the first visit may alter the patient's responses at the second session which is called “carryover effect”. To avoid carryover effect and eliminate the impact of children sensitization to injection on the outcomes, 17 children received routine IANB and 18 patients received IANB + bubble blower BE at the first treatment session and the other treatment was performed for the contralateral quadrant at the second visit. This design may eliminate the impact of such confounding factors [23].

BE acts as a behavioral coping technique and older children are more capable to use behavioral coping strategies than younger children. In addition, children younger than 5 years have insufficient cognitive development to rate pain levels and they have a great tendency for dichotomous and exaggerated rating of self-report scales, which result in invalid interpretations [24]. Thus, 7–10-year-old children were enrolled into the trial. Children of this age group can easily perform the BE with a bubble blower and follow the dentist's orders during IANB injection.

The split-mouth and crossover design of the trial eliminated any possible confounding factors such as parenting style, emotional and cognitive development, and social and economic status. Other confounding factors like parental anxiety or general psychologic disorders were also considered as exclusion criteria during participants recruitment [25].

Girls tend to exhibit increased anxiety in dental setting; gender distribution was 51% girls and 49% boys. Only 45% of the patients had a history of previous dental treatment.

Children with moderate to severe anxiety were enrolled into the trial because anxious children use more coping mechanisms and apply different types of strategies. The anxiety may make them actively deal with the stressful events so they begin to use new strategies [26].

A multidimensional pain and anxiety assessment was performed in the present study and results of subjective and objective evaluation of pain and anxiety were equivalent.

Wilcoxon Signed Rank Test showed no significant differences in FIS between bubble blower and control side (*P* value >0.05). The mean FIS was lower in bubble blower group. These results are in agreement with a study by Sridhar et al. [17]. FIS provides an immediate assessment of children feelings in dental setting. FIS takes less than 1 minute to rate, it has a strong correlation with Venham Picture Test, but it is more simple and more practical to use. However, it is a single-item scale not assessing other anxiety contributing factors; also the momentary mood of the children may not be exactly correlated with their state dental anxiety [27]. To compensate such disadvantages, physiologic parameters assessed the anxiety levels.

Wong–Baker Facial Pain scale is a subjective tool for measuring pain intensity and the most preferred self-report scale by children at any age. It has got adequate psychometric properties and excellent validity [21]. There were significant differences in the mean WBFPS between the test and control sides (*P* value <0.05). These results are in agreement with the results of Sridhar et al. They found significant differences in WBFPS between groups [17]. A recent crossover clinical trial by Omidpanah et al. reported insignificant reductions in the mean of Visual Analogue Scale after BE during buccal infiltration of maxillary canines. They only assessed self-reported pain of the patients and other aspects of the pain and anxiety were not evaluated [28]. A potential disadvantage of WBFPS is the interfering impact of emotions such as tears or smile represented by the faces which may result in higher scores [29]. Therefore, pulse rate, blood pressure, and FLACC scale were administered for objective pain and anxiety assessment [11].

Data analysis did not show any significant differences in the mean of pulse rate and blood pressure between visits (*P* value >0.05); however, the means of both parameters were lower for bubble blower side. Azher et al. compared tell-show-do technique and breathing exercise with a bubble blower during restorative treatments. They showed an increase in the mean pulse rate in bubble blower and a drop in tell-show-do groups [30]. Sridhar et al. revealed higher mean of pulse rate in the bubble blower group compared with the control group but the difference was insignificant [17]. A recent study suggested a significant reduction in office pulse rate and blood pressure after deep breathing exercise. Large inspirations result in lung expansion and activation of pulmonary stretch receptors, stimulation of vagal activity and baroreceptor reflex, and suppressed sympathetic activity, and as a result arterial dilation and reduction of blood pressure and pulse rate occur [11].

A third-year resident of pediatric dentistry and a 20-year experienced pedodontist rated FLACC scale; interobserver correlation was high (*P* value <0.05). The camera was hidden at both visits to avoid the Hawthorne effect and preventing children from modifying their behavior if they became aware of video recording during injection [31]. Wilcoxon Signed Rank Test showed significant differences in FLACC scales measured by two observers between visits (*P* value <0.05). Sridhar et al. revealed significant reduction in FLACC scale in bubble blower group compared with the control group [17], while Peretz et al. reported insignificant reduction in FLACC scores. They evaluated the effect of BE on pain and anxiety levels during IANB injection. BE was administered immediately before injection and no play tools were used in that study [16].

Girls and children without any previous dental treatment may derive more benefits from BE in dental setting compared with boys or children with previous dental treatments. There are several studies that reported increased anxiety and more behavioral management problems in females. Higher initial levels of anxiety in girls may have increased the impact of BE in the present study. Previous dental treatments may alter the children's thought and feelings about dental procedures [27]. No previous negative attitude in children without previous dental treatment as well as administration of play therapy can improve the dentist-child relation resulting in significant differences between test and control visits.

As the relevance to clinical practices, BE using a bubble blower may be a beneficial option for behavioral management of 7–10-year-old children with moderate to severe anxiety specially for short-term interventions such as local anesthesia injection. As a play therapy, it can improve child-dentist relation. It is safe and inexpensive with a high acceptance from children and parents.

Other forms of distraction such as virtual reality have been investigated recently: a study by Nunna et al. compared virtual reality (VR) glasses with counterstimulation during local anesthesia injection. VR group showed significantly less anxiety compared with counterstimulation but similar pain perception; these differences are related to the different design and interventions [32].

Another clinical trial showed significantly lower pain, anxiety, and fear using VR glasses during blood draw in 5–12-year-old children [33]. Custodio et al. conducted a meta-analysis on the effectiveness of VR during different dental procedures. VR was not effective in improving behavior and reducing pain or anxiety of children during local anesthesia injection of rubber dam placement. Children showed better behavior only during caries removal and dental restorations. Also VR glasses are of high costs and not appropriate for all dental procedures, and some children might feel inconvenient with completely blocked vision [34]. Another recent meta-analysis revealed no significant differences in anxiety, fear, and satisfaction of children when receiving local anesthesia with or without VR glasses, as the studies were too heterogeneous to be pooled [35]. Besides the mentioned disadvantages, these results indicate uncertain efficacy of VR interventions; however, based on the present research, BE effectively reduces the pain during IANB and it has less costs and more acceptance.

The first limitation of the study was the sample size of only 35 patients, due to the inclusion criteria chosen for the patients' recruitment and also home quarantine and lockdown due to the worldwide COVID-19 pandemic. The second limitation was the impossibility of blinding children and investigators after allocation of treatment intervention. This may cause some levels of ascertainment bias in outcomes measurements which was unavoidable.

## 5. Conclusion

Breathing exercise using a bubble blower may be an efficient distraction and relaxation method to decrease pain of 7- to 10-year-old children with moderate to severe anxiety during inferior alveolar nerve block. However, anxiety levels were lower when applying BE, and the differences were not statistically significant.

## Figures and Tables

**Table 1:** The mean values of pulse rate and systolic or diastolic blood pressure.

Clinical parameters	Group	*N*	Mean	Std. deviation	Sig.
Systolic blood pressure, before injection	Bubble blower	35	103.54	13.753	0.312
	Control	35	107.03	11.808	
Systolic blood pressure, after injection	Bubble blower	35	111.74	11.150	0.266
	Control	35	116.29	13.932	
Differences of preinjection and postinjection systolic blood pressures	Bubble blower	35	7.91	7.83	0.537
	Control	35	9.26	9.93	
Diastolic blood pressure, before injection	Bubble blower	35	69.89	9.492	0.970
	Control	35	71.43	9.787	
Diastolic blood pressure, after injection	Bubble blower	35	74.60	7.732	0.281
	Control	35	76.11	9.815	
Differences of preinjection and postinjection diastolic blood pressures	Bubble blower	35	4.71	8.34	0.925
	Control	35	4.69	8.43	
Pulse rate, before injection	Bubble blower	35	98.49	14.506	0.426
	Control	35	96.34	12.480	
Pulse rate, after injection	Bubble blower	35	108.60	13.567	0.574
	Control	35	109.51	13.804	
Differences of preinjection and postinjection pulse rate	Bubble blower	35	9.83	9.86	0.051
	Control	35	13.91	11.76	

**Table 2:** Data of FLACC scale, WBFPS, and FIS.

Scales	Group	*N*	Mean	Std. deviation	Sig.
FLACC1 (first observer)	Bubble blower	35	1.46	1.502	0.025
	Control	35	1.86	1.517	
FLACC2 (second observer)	Bubble blower	35	1.14	1.240	0.004
	Control	35	1.94	1.878	
WBFPS	Bubble blower	35	2.97	3.157	0.026
	Control	35	3.71	3.149	
FIS	Bubble blower	35	3.60	0.950	0.336
	Control	35	3.74	0.847	

## Data Availability

The statistical data (raw data of blood pressure, pulse rate, scores of FLACC scale, FIS, and WBFPS and also demographic data of the participants such as gender, age, and history of previous dental treatment) used to support the findings of this study are available from the corresponding author upon request.
